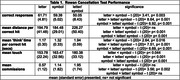# Visual Search Assessed with the Rowan Digital Cancellation Protocol

**DOI:** 10.1002/alz.090091

**Published:** 2025-01-09

**Authors:** Ileana De Anda‐Duran, Lydia Bazzano, Elaine M. Urbina, Jessica Woo, Ganesh Baliga, David J. Libon

**Affiliations:** ^1^ Tulane School of Public Health and Tropical Medicine, New Orleans, LA USA; ^2^ Tulane University School of Public Health and Tropical Medicine, New Orleans, LA USA; ^3^ Cincinnati Children’s Hospital Medical Center & the University of Cincinnati, Cincinnati, OH USA; ^4^ Rowan University, Glassboro, NJ USA; ^5^ Rowan University, Stratford, NJ USA

## Abstract

**Background:**

With the advent of monoclonal antibody therapy to treat mild cognitive impairment and mild dementia due to Alzheimer’s disease (AD) there is a need to develop tests to screen for neurocognitive difficulty that are reliable and easily deployed.

**Method:**

The Rowan Digital Cancellation Tests (RDCT) is comprised of three tests administered using an iPad Pro. Each test was preceded by a practice trial. During practice and test trials a buzzer sounded when commission errors were made. For each test, participants worked for 180 sec. Sixteen targets were located in each quadrant. Target items were embedded within a random array modeled after Weintraub (2000). The Digital Letter Cancellation Test asked participants to circle the letter “A”. The target for the Digital Symbol Cancellation was a geometric symbol. On the Digital Letter/Symbol Switching Cancellation test participants alternated first circling a specific letter, then a specific symbol. Five outcome variables were compiled including correct hits (range 0‐64), distance per correct hit; mean non‐motor/‘think’ time/hit; mean Apple pencil touch; and mean commission errors.

**Result:**

A group of 21 community‐dwelling participants were assessed (age = 51.9±7.2; education = 14.9±2.0; White = 66%, female = 91.5%). A graded pattern of performance was seen for most outcome variables (Table 1) such that more targets were identified for letter>symbol>letter/symbol (p< 0.003), and mean distance traveled per target was less for letter<letter/symbol and symbol< letter/symbol (p< 0.001). Non‐motor/‘think’ time and mean Apple pencil touch increased contingent on task complexity letter<symbol<letter/symbol (p< 0.016, & p< 0.004, respectively). More commission errors emerged on the letter/symbol vs letter test condition (p< 0.002).

**Conclusion:**

Among this sample of community dwelling, generally healthy participants, the RDCT was well‐tolerated. Preliminary data generally yielded graded pattern of performance based on test complexity. When brought to scale these tests could provide a reliable method to screen for neurocognitive decline among patients with neurodegenerative illness.